# Aortic Stent Graft Treatment in a Medium-Size Aortic Center Performed by a Cardiac Surgeon Only—The 9 Years Experience in Poland

**DOI:** 10.3390/jcm13216517

**Published:** 2024-10-30

**Authors:** Marian Burysz, Jakub Batko, Krzysztof Greberski, Artur Słomka, Radosław Litwinowicz

**Affiliations:** 1 Department of Cardiac Surgery, Regional Specialist Hospital, 86-300 Grudziądz, Poland; 2 Thoracic Research Centre, Collegium Medicum Nicolaus Copernicus University, Innovative Medical Forum, 85-094 Bydgoszcz, Poland; 3 Department of Anatomy, Jagiellonian University Medical College, 30-688 Krakow, Poland; 4 CAROL—Cardiothoracic Anatomy Research Operative Lab, Department of Cardiovascular Surgery and Transplantology, Institute of Cardiology, Jagiellonian University Medical College, 30-688 Krakow, Poland; 5 Department of Cardiac Surgery, J. Strus Municipal Hospital, 61-285 Poznan, Poland; 6 Faculty of Health Sciences, University of Medical Sciences, 60-572 Poznan, Poland; 7 Department of Pathophysiology, Nicolaus Copernicus University in Toruń, Ludwik Rydygier Collegium Medicum in Bydgoszcz, 85-094 Bydgoszcz, Poland; 8 National Medical Institute of the Ministry of Interior and Administration, 02-507 Warsaw, Poland

**Keywords:** TEVAR, thoracic aortic aneurysm, stent graft, endovascular treatment, aortic rupture, aortic trauma

## Abstract

**Background**: The incidence of thoracic aortic aneurysms is estimated at 3.0–8.3/100,000 persons per year. There is a lack of reports in the literature on the outcomes of small- and medium-sized thoracic endovascular aortic repairs. The aim of this study is to present the results of thoracic endovascular aortic repairs at a single medium-sized center performed exclusively by a cardiac surgeon. **Methods**: Ninety patients who had undergone aortic stent graft implantations for the treatment of thoracic aortic anomalies were comprehensively, retrospectively evaluated. The detailed preoperative, surgical, and postoperative parameters of the patients, including the survival rate up to five years, were recorded and further analyzed. **Results**: The patients’ Euroscores were four (2.1–9). The 30-day mortality rate was 8.9%, the 1-year mortality rate was 15.6%, and the 5-year mortality rate was 38.9% for all causes. Postoperative complications were observed in 10% of the patients. Statistically significant differences were observed between the urgency of surgery at 30 days and survival at one year, but not at five years. The most common complications were related to respiratory (4.4%), renal (3.3%), and neurological (3.3%) dysfunction. **Conclusions**: Thoracic endovascular aortic repair can be safely performed in small- and medium-sized centers with optimal long-term results.

## 1. Introduction

Thoracic aortic aneurysms incidence is estimated to occur in 3.0–8.3/100,000 individuals per year [1]. It may not be associated with any specific symptoms, thus leading to the rupture associated mortality of up to 90%. Ruptures are observed in 1.3–2.1/100,000 individuals per year [1]. The American Heart Association in their most recent guidelines underlines the importance of the thoracic endovascular aortic repair in patients with an aortic aneurysm rupture [2]. Additionally, it should be noted that endovascular procedure implementation in the treatment of thoracic aortic pathologies grows exponentially with the use of commercially available or customized aortic stent grafts [3].

Thoracic endovascular aortic repair was introduced and performed for the first time in 1987 by Dr. Nikolay Volodos in Ukraine [4]. This procedure provides a safe and effective approach for the treatment of aortic pathology located below the aortic arch, including an aortic aneurysm, an intramural hematoma, and a penetrating aortic ulcer or traumatic aortic injury. It includes the visualization of the pathologically altered aorta, the implantation of a stent graft, and the final confirmation of the correct location of the device without the presence of endoleaks. The procedure is performed exclusively via vascular access, so that a sternotomy can be avoided. The stent graft implantation can be performed as a second stage after the implantation of a frozen elephant trunk for pathologies of the aortic arch. The most common complications of the procedure are a progression of aortic disease, spinal cord ischemia, negative cardiac remodeling, and endoleaks [5,6,7,8,9,10,11,12,13,14,15].

There is a lack of reports in the literature on the outcomes of small- and medium-sized thoracic endovascular aortic repairs, which may be helpful for future development and improved access to this procedure for a broader patient population.

The aim of this study is to present detailed results of thoracic endovascular aortic repairs at a single mid-sized center performed exclusively by a cardiac surgeon, including detailed information on the postoperative outcomes based on the indication for the procedure, urgency, and patient gender.

## 2. Materials and Methods

### 2.1. Patients’ Characteristics

All patients who underwent an aortic stent graft implantation for treatment of thoracic aorta abnormalities between 1 May 2015 and 1 May 2024 at the Regional Specialized Hospital in Grudziadz, Poland, were comprehensively analyzed retrospectively. Patients’ demographic characteristics, preoperative comorbidities, intervention indications, intervention urgency, and detailed surgical and postoperative parameters, including up to five years survivability, were collected and further analyzed. The 30-day, 1-year, and 5-year mortality rates were collected from the National Health Fund, the obligatory public health insurance institution in Poland, and incorporated into the KROK (Polish National Registry of Cardiac Surgery Procedures) registry (available at: https://krok.csioz.gov.pl) on 1 August 2024. Due to the retrospective nature of this study, the approval of the Bioethics Committee was waived. This study’s protocol complies with the ethical guidelines of the Declaration of Helsinki of 1975.

### 2.2. Procedure

Briefly, all thoracic endovascular aortic repair procedures at our institution are conducted under general anesthesia in a hybrid operating room, utilizing a C-arm fluoroscope. The patient is positioned with their groin, abdomen, and chest exposed. The right femoral artery is the preferred access route for the procedure. The femoral artery is surgically exposed under direct visualization, followed by the placement of a Prolene 6.0 suture. Access is established using a standard 5 Fr sheath. The patient is then heparinized to achieve an activated clotting time of 200 s. A pigtail catheter is introduced via the femoral or brachial/radial artery to perform an aortogram of the area of interest.

After the angiogram, the aneurysm is evaluated, with the length and diameter of the proximal and distal neck measured using both the preoperative computed tomography scan and the angiogram. Through femoral access, a diagnostic catheter is advanced and subsequently exchanged for extra stiff wire guides. Based on these measurements, the appropriate stent graft is selected, flushed with heparinized solution, and advanced to the proximal neck. If necessary, a repeat angiogram is performed to reconfirm the positioning of the device within the aorta and the landing zone. Before deploying the device, rapid pacing through the jugular vein is performed to ensure precise deployment and prevent migration due to forward arterial blood flow. After deployment, a completion angiogram is conducted to confirm the absence of a gross endoleak. At this point, the stent graft may be ballooned to reduce the risk of Type I or III endoleaks.

### 2.3. Definitions

We defined a small-sized aortic center as a center performing less than 15 procedures on a thoracic aorta annually. We defined a medium-sized aortic center as a center performing more than 15 procedures and less than 30 procedures on a thoracic aorta annually. We defined a large-sized aortic center as a center performing more than 30 procedures on a thoracic aorta annually. Our center fits the definition of the medium-sized aortic center. This division was inspired by the 2022 ACC/AHA aortic treatment guidelines [2].

### 2.4. Statistical Analysis

Data were analyzed using IBM SPSS Statistics 29.0 (Predictive Solutions, Pittsburgh, PA, USA). Categorical variables are presented as numbers (*n*) or percentages. Quantitative variables are presented as the median with first and third quartiles. The normal distribution was analyzed using the Shapiro–Wilk test. A continuous variables simple group comparison was performed with the U-Mann–Whitney test. A continuous variables multi-group comparison was assessed using the Kruskal and Wallis test with the Dunn’s post hoc test with Bonferroni correction if the results of the Kruskal and Wallis test were statistically significant. For the categorical variables, the chi-square test for independence or Fischer’s exact test was used. Survival curves were performed for all patients, with an additional analysis including the following subgroups: sex, intervention urgency, and intervention indications. A *p*-value < 0.05 was considered statistically significant.

## 3. Results

### 3.1. Characteristics of the Patients

Between 1 May 2015 and 1 May 2024, 90 patients (median age: 64 years (55–70), with 72.2% male) were admitted to our hospital and underwent an aortic stent graft implantation.

#### 3.1.1. Characteristics of the Patients—Sex Comparison

A comparison of the detailed preoperative characteristics of the patients based on sex are presented in Table 1.

#### 3.1.2. Characteristics of the Patients—Surgery Urgency

A comparison of the detailed preoperative characteristics of the patients with surgery urgency is presented in Table A1 in Appendix A.

Statistically significant differences were observed between the groups with hypertension (the post-hoc comparison was significantly different between acute and chronic aortic dissection), peripheral vascular disease (the post-hoc comparison was significantly different between acute aortic dissection and aortic aneurysm), and with poor mobility (the post-hoc comparison was significantly different between acute aortic dissection and aortic aneurysm).

#### 3.1.3. Characteristics of the Patients—Surgery Indication

A comparison of the detailed preoperative characteristics of the patients with surgery indication is presented in Table A2 in Appendix A. Statistically significant differences were observed between the groups with hypertension (the post-hoc comparison was significantly different between acute and chronic aortic dissection), with peripheral vascular disease (the post-hoc comparison was significantly different between acute aortic dissection and aortic aneurysm), and with poor mobility (the post-hoc comparison was significantly different between acute aortic dissection and aortic aneurysm).

### 3.2. Intraoperative and Postoperative Outcomes

#### 3.2.1. Intraoperative and Postoperative Outcomes—Sex Comparison

**Figure 1:**
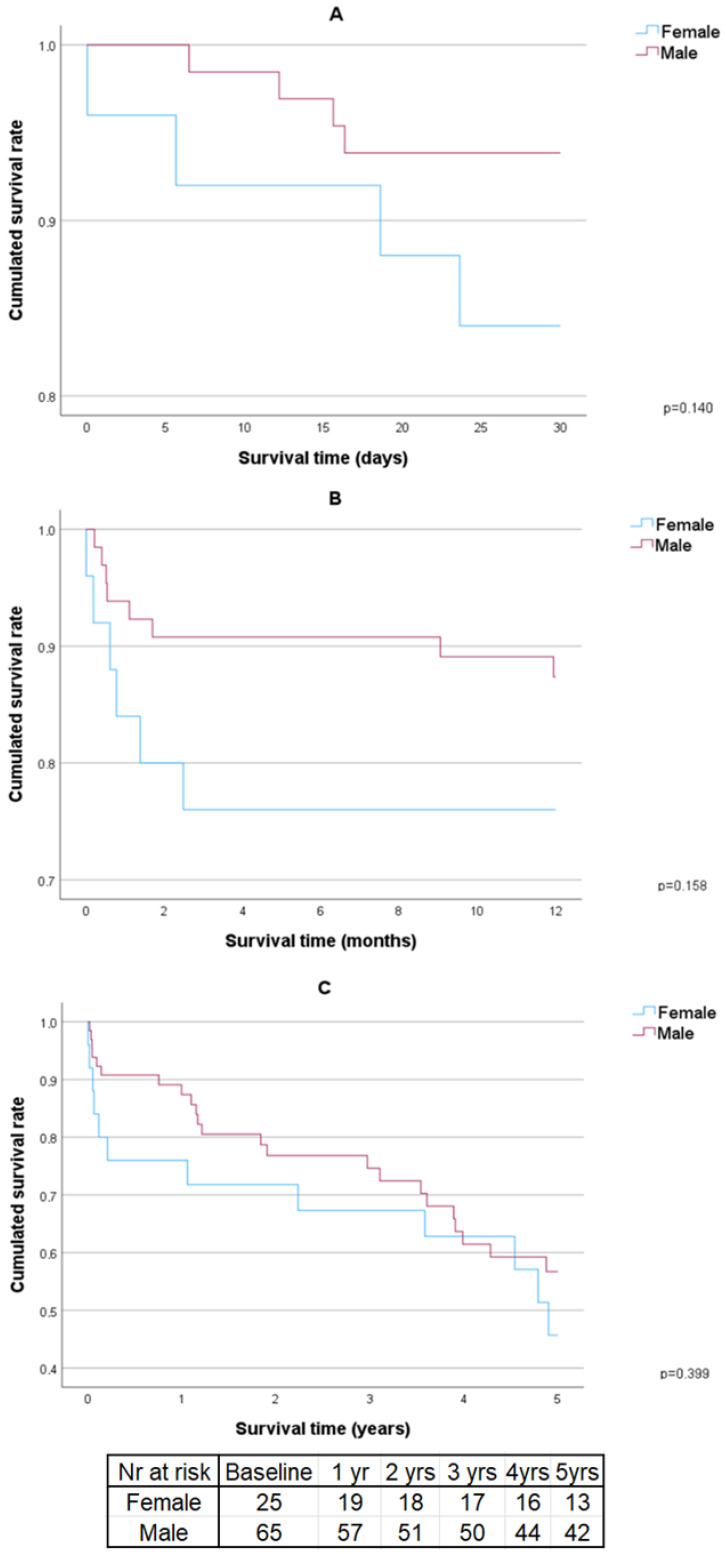
Survival curves with sex comparison. (**A**) 30 days survival curve, (**B**) 1 year survival curve, (**C**) 5 years survival curve. Yr—year.

A comparison of the detailed intraoperative and postoperative outcomes for males and females can be found in Table 2. The 30-day, 1-year and 5-year survival curves with a sex comparison can be found in Figure 1A–C.

Significant differences were observed only in the Euroscores (significantly larger in females). No statistically significant differences were observed between the sexes in relation to 30-day, 1-year and 5-year survivability.

#### 3.2.2. Intraoperative and Postoperative Outcomes—Surgery Urgency

**Figure 2:**
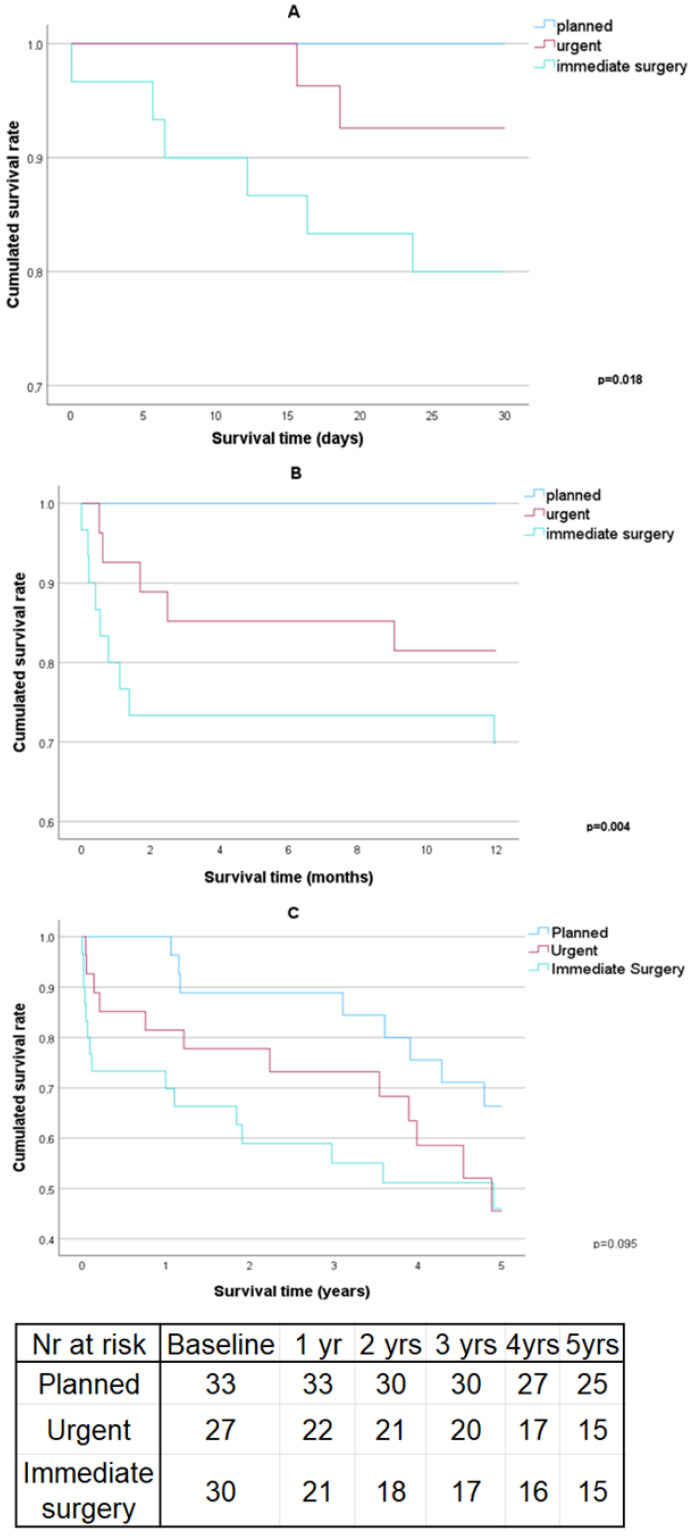
Survival curves with surgery urgency comparison. (**A**) 30-day survival curve, (**B**) 1-year survival curve, (**C**) 5-year survival curve. Yr—year.

The detailed intraoperative and postoperative outcomes with a surgery urgency comparison can be found in Table 3. The 30-day, 1-year and 5-year survival curves with a surgery urgency comparison can be found in Figure 2A–C.

Significant differences were observed in the Euroscores (significantly lower in the planned procedures vs. the urgent and immediate surgeries), surgery indication, and intubation time (significantly longer in immediate surgeries). Statistically significant differences were observed between surgery urgency in relation to the 30-day and 1-year survivability rates; however, it was not observed in the 5-year survivability rate.

#### 3.2.3. Intraoperative and Postoperative Outcomes—Surgery Indication

**Figure A1:**
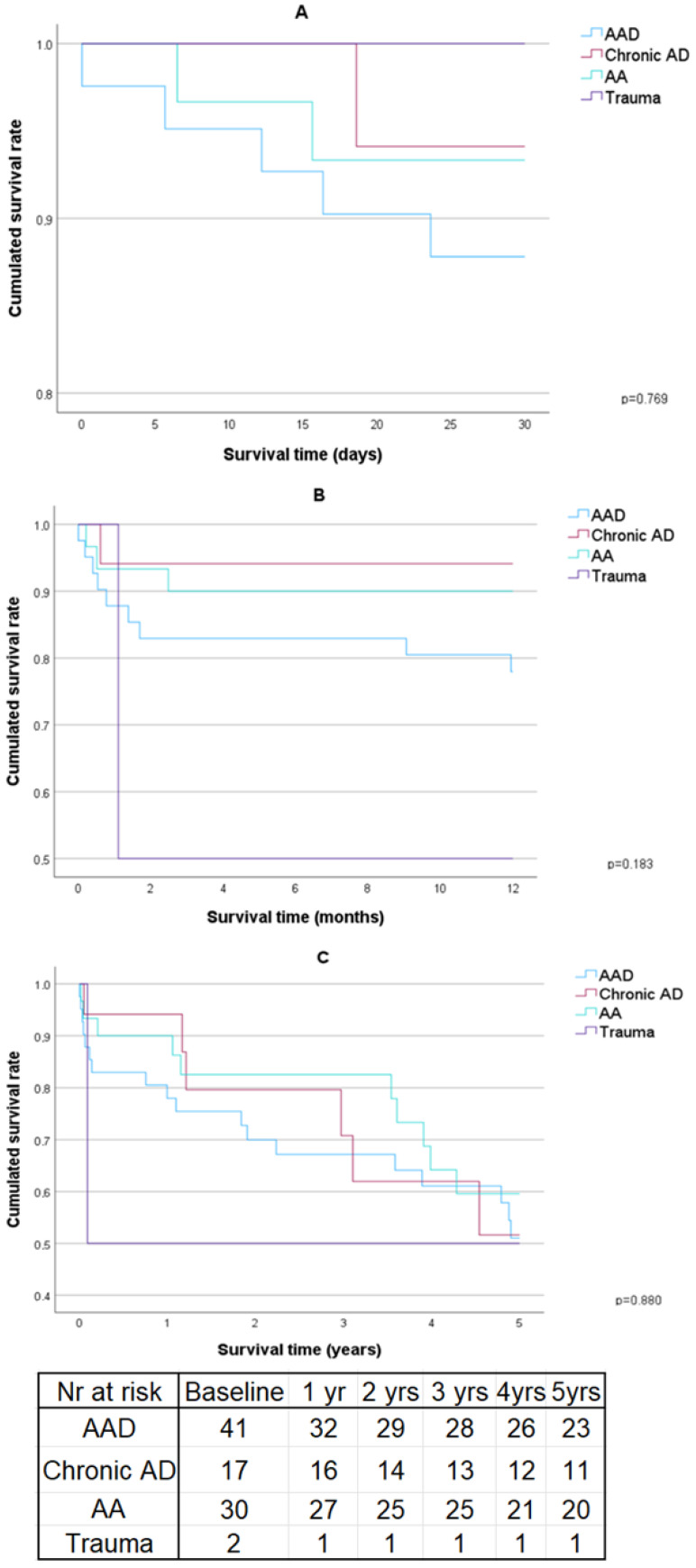
Survival curves with a surgery indication comparison. (**A**) 30-day survival curve, (**B**) 1-year survival curve, (**C**) 5-year survival curve. AAD—acute aortic dissection, AD—aortic dissection, AA—aortic aneurysm.

The detailed intraoperative and postoperative outcomes with a surgery indication comparison can be found in Table A3. The 30-day, 1-year, and 5-year survival curves with a surgery indication comparison can be found in Figure A1A–C.

Significant differences were observed in Euroscores (significantly larger in acute aortic dissections vs. chronic aortic dissections and aortic aneurysms), procedure urgency (immediate surgery was most commonly in acute aortic dissections), and postoperative transfusion (least common in aortic aneurysms). No statistically significant differences were observed between the surgery indications in relation to the 30-day, 1-year, and 5-year survivability rates.

## 4. Discussion

### 4.1. Results Discussion

An analysis of the outcomes of the thoracic endovascular aortic repairs in our population revealed a 30-day and 1-year mortality of 8.9% and 15.6%, respectively, which should be considered great, especially with a 63.3% rate of urgent surgery and a comparable mortality rate previously reported in the literature for large aortic centers [16]. The five-year mortality rate of 38.9% should be interpreted with caution as the exact cause of death of the patients is unknown. Only one case required reoperation due to an endoleak, which establishes a prevalence at 1.1%, compared to 9.5% in the literature [6]. There were no significant differences between women and men in the 30-day, 1-year, and 5-year observations. It is especially important in regard to patient qualification, as patients should not be taken into account as an additional risk factor in such a procedure.

It should be noted that in patients grouped based on procedure urgency, a significant difference was observed in age and hypertension—especially untreated, peripheral vascular disease—which was mostly observed in patients that qualified for an urgent procedure. In those populations, the main differences were observed in the 30-day and 1-year mortality rates, with no significant difference in the 5-year mortality rate, which proves that aortic disease, especially its aneurysm or dissection, increases the long-term mortality in all patients. However, a planned character for the procedure is the most optimal approach, and if possible it should be performed in every patient with aortic pathology, as procedure urgency increases intraoperative and postoperative mortality.

Recently, we have introduced sedation as the main anesthetic procedure for stent graft implantation. However, due to the small number of patients (10), it is still too early to assess the long-term benefits of such a procedure. We achieved a shorter operation time (90 vs. 154.2 min) and a shorter stay in the intensive care unit (0.9 vs. 1.95 days) than in the previously published study [17].

We observed complications in 10% of the patients. The most common complications, including respiratory (4.4%) and renal (3.3%) complications, were related to the critical preoperative condition of the patients. In three patients, we observed neurological complications, including spinal cord ischemia (2 cases) and transient ischemic attack (one case), at a rate similar to previous studies [10,11,12,13].

### 4.2. Thoracic Endovascular Aortic Repair Indications

#### 4.2.1. Acute Aortic Dissection

The urgent treatment of acute aortic dissection is required in patients with diagnosed malperfusion, persistent pain, unstable or rapid hypertension, and a radiologically confirmed extension of the dissection. General indications for thoracic endovascular aortic repair for subacute aortic dissection include a total aortic diameter greater than 40 mm, a false lumen diameter greater than 25 mm, a primary entry tear greater than 10 mm, and an entry tear communication in the internal aortic curvature [18].

#### 4.2.2. Descending Aortic Aneurysms

Thoracic endovascular aortic repair should be performed in patients with an aneurysm larger than 55 mm, although this may be lower in patients with connective tissue disorders such as Marfan syndrome or in women. The procedure should be performed in patients with a rapidly growing aneurysm, which is defined as growth rate of more than 10 mm/year [2,18,19].

#### 4.2.3. Intramural Hematomas and Penetrating Aortic Ulcers

According to the most recent guidelines, penetrating aortic ulcers with a depth of more than 10 mm and a diameter of more than 20 mm are an indication of the need for thoracic endovascular aortic repair. It should be noted that patients with intramural hematomas that occur concomitantly with an aortic ulcer require more frequent follow-up [20].

#### 4.2.4. Traumatic Aortic Injuries

For traumatic aortic injuries, thoracic endovascular aortic repair should be considered first, as it is less invasive and provides excellent results [21]. Even penetrating aortic trauma with a penetrating factor remaining in the aortic lumen can be successfully treated in this way [22].

### 4.3. Preoperative Imaging

The gold standard for aortic imaging in patients with a suspected or confirmed pathology of the thoracic aorta is electrocardiography-guided, contrast-enhanced computed tomography of the entire aorta [2,19]. It enables the correct measurement of the aorta, which is necessary for the adjustment of the stent graft, the assessment of the entry site and the vessels involved in aortic pathology, and provides additional information on the possible restrictions to vascular access. It also provides detailed information about the patient’s vascular anatomy, which may be helpful for future interventions in this region [23,24].

### 4.4. Postoperative Aftercare

Strict follow-up care is required to achieve good early and long-term results. Great care must be taken during the short-term follow-up and during hospitalization to detect an early air embolism or other ischemic complications that may be iatrogenic [16,25]. A computed tomographic angiography is recommended at 6 and 12 months postoperation and then annually. Regular imaging helps to detect late complications such as progression of aortic disease, including a type A retrograde aortic dissection, or endoleaks [6,7,8,9,10,11,12,13,14,17,20,25]. Left ventricular fraction and blood pressure should be closely monitored as there are previous reports of adverse cardiac remodeling with a decreased ejection fraction and increased blood pressure in patients undergoing thoracic endovascular aortic repair [13]. We did not observe such changes in our patients.

### 4.5. Limitations

This is a retrospective, observational study, the results of which should be interpreted with caution. We did not receive complete information regarding mortality causes, which may be connected with the lower rates of cardiac-associated mortality. We did not collect information regarding patients’ quality of life postoperation. Future studies should focus on refining the risk stratification tools, especially in identifying high-risk patients for a tailored management.

## 5. Conclusions

Thoracic endovascular aortic repair can be safely performed in small- and medium-sized centers with optimal long-term results.

## Figures and Tables

**Table 1:** Preoperative characteristics patients, a comparison based on sex. BMI—body mass index, TIA—transient ischemic attack, GFR—glomerular filtration rate.

		Female (*n* = 25)	Male (*n* = 65)	General	*p*
Age (years)		66 (59–73)	63 (55–68)	64 (55–70)	0.168
BMI (kg/m^2^)		29.4 (26–33.7)	26.6 (24.5–30.8)	27 (24.7–32.7)	0.340
CCS Class	1	15 (60%)	48 (73.8%)	63 (15.6%)	0.346
	2	9 (36%)	13 (20%)	22 (24.4%)	
	3	1 (4%)	2 (3.1%)	3 (3.3%)	
	4	0 (0%)	2 (3.1%)	2 (2.2%)	
NYHA class	1	12 (48%)	44 (67.7%)	56 (11.1%)	0.481
	2	8 (32%)	15 (23.1%)	23 (25.6%)	
	3	2 (8%)	2 (3.1%)	4 (4.4%)	
	4	3 (12%)	4 (6.2%)	7 (7.8%)	
Eversmoker	actual	6 (24%)	19 (29.2%)	25 (27.8%)	0.853
	previous	11 (44%)	25 (38.5%)	36 (40%)	
Diabetes mellitus type 2	diet	0 (0%)	1 (1.5%)	1 (1.1%)	0.134
	pharmacological	3 (12%)	2 (3.1%)	5 (5.6%)	
	insulin	4 (16%)	4 (6.2%)	8 (8.9%)	
Hypertension	treated	19 (76%)	47 (72.3%)	66 (73.3%)	0.488
	untreated	4 (16%)	7 (10.8%)	11 (12.2%)	
Hyperlipidemia		10 (40%)	23 (35.4%)	33 (36.7%)	0.684
TIA		1 (4%)	2 (3.1%)	3 (3.3%)	0.239
Peripheral vascular disease		11 (44%)	17 (26.2%)	28 (31.1%)	0.248
Renal impairment	GFR > 85	13 (52%)	35 (53.8%)	48 (53.3%)	0.067
	50 < GFR < 86	5 (20%)	24 (36.9%)	29 (32.2%)	
	GFR < 50	6 (24%)	6 (9.2%)	12 (13.3%)	
	dialysis	1 (4%)	0 (0%)	1 (1.1%)	
Poor mobility		10 (40%)	15 (23.1%)	25 (27.8%)	0.108
Chronic lung disease		3 (12%)	4 (6.2%)	7 (7.8%)	0.354
Critical preoperative condition		7 (28%)	15 (23.1%)	22 (24.4%)	0.626
Preoperative mechanical ventilation		0 (0%)	5 (7.7%)	5 (5.6%)	0.317
Cardiogenic shock		2 (8%)	7 (10.8%)	9 (10%)	1.000
Previous thoraflex implantation		0 (0%)	6 (9.2%)	6 (6.7%)	0.181
Time from thoraflex implantation (months)		0 (0–0)	2.5 (1.6–4.1)	2.5 (1.6–4.1)	-

No significant differences were observed in sex comparison.

**Table 2:** Comparison of intraoperative and postoperative outcomes of patients’ based on sex. ICU—intensive care unit. Significant *p* values bolded.

		Female (*n* = 25)	Male (*n* = 65)	General	*p*
Euroscore		6.7 (3.7–16.8)	3.5 (1.7–7.2)	4 (2.1–9)	**0.011**
Procedure urgency	planned	6 (24%)	27 (41.5%)	33 (36.7%)	0.254
	urgent	10 (40%)	17 (26.2%)	27 (30%)	
	immediate surgery	9 (36%)	21 (32.3%)	30 (33.3%)	
Surgery indication	acute aortic dissection	13 (52%)	28 (43.1%)	41 (45.6%)	0.740
	chronic aortic dissection	4 (16%)	13 (20%)	17 (18.9%)	
	aortic aneurysm	8 (32%)	22 (33.8%)	30 (33.3%)	
	penetrating trauma	0 (0%)	2 (3.1%)	2 (2.2%)	
Aortic segments involved	thoracic descending	17 (68%)	48 (73.8%)	65 (72.2%)	0.854
	thoracic and abdominal	2 (8%)	4 (6.2%)	6 (6.7%)	
	distal arch and thoracic	6 (24%)	13 (20%)	19 (21.1%)	
Type of anesthesia	general	21 (84%)	59 (90.8%)	80 (89.9%)	0.698
	sedation	3 (12%)	6 (9.2%)	9 (10.1%)	
Surgery time (min)		95 (80–120)	90 (70–120)	90 (70–120)	0.658
Intubation time (h)		5.7 (3.3–8.3)	3.5 (1.8–8.3)	3.9 (1.9–8.3)	0.152
Postoperative transfusion		7 (28%)	25 (38.5%)	32 (35.6%)	0.353
ICU stay (days)		0.9 (0.2–1.1)	0.8 (0.1–1.1)	0.9 (0.1–1.1)	0.432
Hospitalization time (days)		9 (6–12.5)	7 (5–11.5)	7.5 (5–11.5)	0.321
30 days mortality		4 (16%)	4 (6.2%)	8 (8.9%)	0.211
1 year mortality		6 (24%)	8 (12.3%)	14 (15.6%)	0.200
5 years mortality		12 (48%)	23 (35.4%)	35 (38.9%)	0.272
Postoperative complications		3 (12%)	6 (9.2%)	9 (10%)	0.695
Reoperation		0 (0%)	1 (1.5%)	1 (1.1%)	-
Fresh miocardial infarction		0 (0%)	1 (1.5%)	1 (1.1%)	-
Hemodialysis		0 (0%)	1 (1.5%)	1 (1.1%)	-
Respiratory system complications		1 (4%)	3 (4.6%)	4 (4.4%)	1.000
Renal complications		1 (4%)	2 (3.1%)	3 (3.3%)	1.000
Neurological complications		2 (8%)	1 (1.5%)	3 (3.3%)	0.186
Tamponade		1 (4%)	2 (3.1%)	3 (3.3%)	1.000

**Table 3:** Intraoperative and postoperative outcomes for patients with a comparison based on surgery urgency. ICU—intensive care unit.

		Planned (*n* = 33)	Urgent (*n* = 27)	Immediate Surgery (*n* = 30)	*p*
Euroscore		1.7 (1.3–3.4)	4.5 (3.3–13.8)	7.5 (4.1–16.8)	<0.001
Surgery indication	acute aortic dissection	4 (12.1%)	12 (44.4%)	25 (83.3%)	<0.001
	chronic aortic dissection	9 (27.3%)	6 (22.2%)	2 (6.7%)	
	aortic aneurysm	20 (60.6%)	9 (33.3%)	1 (3.3%)	
	penetrating trauma	0 (0%)	0 (0%)	2 (6.7%)	
Aortic segments involved	thoracic descending	24 (72.7%)	20 (74.1%)	21 (70%)	0.299
	thoracic and abdominal	3 (9.1%)	3 (11.1%)	0 (0%)	
	distal arch and thoracic	6 (18.2%)	4 (14.8%)	9 (30%)	
Type of anesthesia	general	27 (81.8%)	26 (96.3%)	27 (93.1%)	0.141
	sedation	6 (18.2%)	1 (3.7%)	2 (6.9%)	
Surgery time (min)		90 (75–120)	90 (60–120)	95 (69–150)	0.582
Intubation time (h)		2.6 (1.8–6.5)	2.8 (1.3–5.1)	8.3 (3.3–33.9)	0.008
Postoperative transfusion		10 (30.3%)	8 (29.6%)	14 (46.7%)	0.297
ICU stay (days)		0.9 (0–1.1)	0.9 (0.1–1.1)	0.9 (0.4–3)	0.115
Hospitalization time (days)		7 (5–9)	11 (6–15)	7 (4–11.5)	0.196
30 days mortality		0 (0%)	2 (7.4%)	6 (20%)	0.020
1 year mortality		0 (0%)	5 (18.5%)	9 (30%)	0.004
5 years mortality		8 (24.2%)	12 (44.4%)	15 (50%)	0.087
Postoperative complications		1 (3%)	3 (11.1%)	5 (16.7%)	0.192
Reoperation		1 (3%)	0 (0%)	0 (0%)	-
Fresh miocardial infarction		0 (0%)	0 (0%)	1 (3.3%)	-
Hemodialysis		0 (0%)	0 (0%)	1 (3.3%)	-
Respiratory system complications		0 (0%)	2 (7.4%)	2 (6.7%)	-
Renal complications		0 (0%)	0 (0%)	3 (10%)	-
Neurological complications		0 (0%)	0 (0%)	3 (10%)	-
Tamponade		1 (3%)	1 (3.7%)	1 (3.3%)	0.990

## Data Availability

Data are available from the corresponding author upon reasonable request.

## Appendix A

**Table A1:** Preoperative characteristics of patients, a comparison based on surgery urgency. BMI—body mass index, TIA—transient ischemic attack, GFR—glomerular filtration rate.

		Planned (*n* = 33)	Urgent (*n* = 27)	Immediate Surgery (*n* = 30)	*p*
Age (years)		63 (56–68)	67 (61–74)	58.5 (53–66)	0.036
Male		27 (81.8%)	17 (63%)	21 (70%)	0.254
BMI (kg/m^2^)		28.1 (25.9–33.6)	26.5 (24.1–33.7)	26.6 (24.2–30.7)	0.254
CCS Class	1	26 (21.2%)	17 (7.4%)	20 (16.7%)	0.622
	2	6 (18.2%)	8 (29.6%)	8 (26.7%)	
	3	0 (0%)	2 (7.4%)	1 (3.3%)	
	4	1 (3%)	0 (0%)	1 (3.3%)	
NYHA class	1	22 (6.1%)	15 (7.4%)	19 (20%)	0.281
	2	10 (30.3%)	7 (25.9%)	6 (20%)	
	3	1 (3%)	2 (7.4%)	1 (3.3%)	
	4	0 (0%)	3 (11.1%)	4 (13.3%)	
Eversmoker	actual	5 (15.2%)	8 (29.6%)	12 (40%)	0.191
	previous	14 (42.4%)	10 (37%)	12 (40%)	
Diabetes mellitus type 2	diet	0 (0%)	0 (0%)	1 (3.3%)	0.067
	pharmacological	2 (6.1%)	2 (7.4%)	1 (3.3%)	
	insulin	2 (6.1%)	6 (22.2%)	0 (0%)	
Hypertension	treated	27 (81.8%)	24 (88.9%)	15 (50%)	<0.001
	untreated	0 (0%)	2 (7.4%)	9 (30%)	
Hyperlipidemia		9 (27.3%)	14 (51.9%)	10 (33.3%)	0.130
TIA		0 (0%)	3 (11.1%)	0 (0%)	-
Peripheral vascular disease		7 (21.2%)	14 (51.9%)	7 (23.3%)	0.001
Renal impairment	GFR > 85	20 (60.6%)	10 (37%)	18 (60%)	0.102
	50 < GFR < 86	12 (36.4%)	11 (40.7%)	6 (20%)	
	GFR < 50	1 (3%)	6 (22.2%)	5 (16.7%)	
	dialysis	0 (0%)	0 (0%)	1 (3.3%)	
Poor mobility		1 (3%)	6 (22.2%)	18 (60%)	<0.001
Chronic lung disease		1 (3%)	3 (11.1%)	3 (10%)	0.436
Critical preoperative condition		0 (0%)	5 (18.5%)	17 (56.7%)	-
Preoperative mechanical ventilation		0 (0%)	0 (0%)	5 (16.7%)	-
Cardiogenic shock		0 (0%)	0 (0%)	9 (30%)	-
Previous thoraflex implantation		6 (18.2%)	0 (0%)	0 (0%)	-
Time from thoraflex implantation (months)		2.5 (1.6–4.1)	0 (0–0)	0 (0–0)	-

**Table A2:** Preoperative characteristics of patients, a comparison based on surgery indication. BMI—body mass index, TIA—transient ischemic attack, GFR—glomerular filtration rate.

		Acute Aortic Dissection (*n* = 41)	Chronic Aortic Dissection (*n* = 17)	Aortic Aneurysm (*n* = 30)	Penetrating Trauma (*n* = 2)	*p*
Age (years)		65 (53–72)	64 (57–68)	63 (59–69)	34.5 (25–44)	0.2
Male		28 (68.3%)	13 (76.5%)	22 (73.3%)	2 (100%)	0.740
BMI (kg/m^2^)		26.5 (24.3–30)	27 (23.7–34.2)	27.6 (26–33.7)	27.5 (24.2–30.7)	0.452
CCS Class	1	25 (12.2%)	13 (17.6%)	23 (16.7%)	2 (50%)	0.883
	2	13 (31.7%)	3 (17.6%)	6 (20%)	0 (0%)	
	3	2 (4.9%)	1 (5.9%)	0 (0%)	0 (0%)	
	4	1 (2.4%)	0 (0%)	1 (3.3%)	0 (0%)	
NYHA class	1	25 (19.5%)	11 (0%)	19 (6.7%)	1 (0%)	0.056
	2	8 (19.5%)	6 (35.3%)	9 (30%)	0 (0%)	
	3	2 (4.9%)	0 (0%)	2 (6.7%)	0 (0%)	
	4	6 (14.6%)	0 (0%)	0 (0%)	1 (50%)	
Eversmoker	actual	11 (26.8%)	6 (35.3%)	7 (23.3%)	1 (50%)	0.287
	previous	21 (51.2%)	6 (35.3%)	9 (30%)	0 (0%)	
Diabetes mellitus type 2	diet	1 (2.4%)	0 (0%)	0 (0%)	0 (0%)	0.795
	pharmacological	1 (2.4%)	2 (11.8%)	2 (6.7%)	0 (0%)	
	insulin	2 (4.9%)	2 (11.8%)	4 (13.3%)	0 (0%)	
Hypertension	treated	26 (63.4%)	13 (76.5%)	26 (86.7%)	1 (50%)	0.03
	untreated	10 (24.4%)	0 (0%)	1 (3.3%)	0 (0%)	
Hyperlipidemia		16 (39%)	7 (41.2%)	10 (33.3%)	0 (0%)	-
TIA		0 (0%)	1 (5.9%)	2 (6.7%)	0 (0%)	-
Peripheral vascular disease		20 (48.8%)	4 (23.5%)	3 (10%)	1 (50%)	0.024
Renal impairment	GFR > 85	20 (48.8%)	10 (58.8%)	17 (56.7%)	1 (50%)	0.874
	50 < GFR < 86	12 (29.3%)	5 (29.4%)	11 (36.7%)	1 (50%)	
	GFR < 50	8 (19.5%)	2 (11.8%)	2 (6.7%)	0 (0%)	
	dialysis	1 (2.4%)	0 (0%)	0 (0%)	0 (0%)	
Poor mobility		17 (41.5%)	3 (17.6%)	3 (10%)	2 (100%)	0.002
Chronic lung disease		6 (14.6%)	0 (0%)	1 (3.3%)	0 (0%)	-
Critical preoperative condition		20 (48.8%)	0 (0%)	1 (3.3%)	1 (50%)	-
Preoperative mechanical ventilation		4 (9.8%)	1 (5.9%)	0 (0%)	0 (0%)	-
Cardiogenic shock		6 (14.6%)	1 (5.9%)	0 (0%)	2 (100%)	-
Previous thoraflex implantation		2 (4.9%)	3 (17.6%)	1 (3.3%)	0 (0%)	-
Time from thoraflex implantation (months)		1.6 (1.6–1.6)	4.1 (3.5–19.6)	1.2 (1.2–1.2)	0 (0–0)	0.11

**Table A3:** Intraoperative and postoperative outcomes of patients, a comparison based on surgery indication. ICU—intensive care unit.

		Acute Aortic Dissection (*n* = 41)	Chronic Aortic Dissection (*n* = 17)	Aortic Aneurysm (*n* = 30)	Penetrating Trauma (*n* = 2)	*p*
Euroscore		8.3 (3.9–25.1)	3.2 (1.6–4.3)	2.5 (1.3–4.1)	5.8 (2.4–9.1)	<0.001
Procedure urgency	planned	4 (9.8%)	9 (52.9%)	20 (66.7%)	0 (0%)	<0.001
	urgent	12 (29.3%)	6 (35.3%)	9 (30%)	0 (0%)	
	immediate surgery	25 (61%)	2 (11.8%)	1 (3.3%)	2 (100%)	
Aortic segments involved	thoracic descending	29 (70.7%)	13 (76.5%)	21 (70%)	2 (100%)	0.983
	thoracic and abdominal	3 (7.3%)	1 (5.9%)	2 (6.7%)	0 (0%)	
	distal arch and thoracic	9 (22%)	3 (17.6%)	7 (23.3%)	0 (0%)	
Type of anesthesia	general	38 (95%)	14 (82.4%)	26 (86.7%)	2 (100%)	0.427
	sedation	2 (5%)	3 (17.6%)	4 (13.3%)	0 (0%)	
Surgery time (min)		90 (69–120)	90 (70–120)	92.5 (70–115)	117.5 (115–120)	0.697
Intubation time (h)		3.8 (1.9–9.2)	5.5 (2–7.7)	3.6 (1.4–5.2)	18.8 (3.5–34.1)	0.313
Postoperative transfusion		18 (43.9%)	8 (47.1%)	4 (13.3%)	2 (100%)	0.006
ICU stay (days)		0.9 (0.2–1.7)	1 (0.4–1.4)	0.6 (0.1–1)	1.5 (0.1–3)	0.340
Hospitalization time (days)		9 (6–12)	8 (6–16)	7 (5–9)	2 (0–4)	0.081
30-day mortality		5 (12.2%)	1 (5.9%)	2 (6.7%)	0 (0%)	-
1-year mortality		9 (22%)	1 (5.9%)	3 (10%)	1 (50%)	0.172
5-year mortality		18 (43.9%)	6 (35.3%)	10 (33.3%)	1 (50%)	0.797
Postoperative complications		7 (17.1%)	1 (5.9%)	1 (3.3%)	0 (0%)	-
Reoperation		0 (0%)	0 (0%)	1 (3.3%)	0 (0%)	-
Fresh miocardial infarction		1 (2.4%)	0 (0%)	0 (0%)	0 (0%)	-
Hemodialysis		1 (2.4%)	0 (0%)	0 (0%)	0 (0%)	-
Respiratory system complications		3 (7.3%)	1 (5.9%)	0 (0%)	0 (0%)	-
Renal complications		3 (7.3%)	0 (0%)	0 (0%)	0 (0%)	-
Neurological complications		3 (7.3%)	0 (0%)	0 (0%)	0 (0%)	-
Tamponade		2 (4.9%)	0 (0%)	1 (3.3%)	0 (0%)	-
